# Human faces and face‐like stimuli are more memorable

**DOI:** 10.1002/pchj.564

**Published:** 2022-06-05

**Authors:** Marianna E. Kapsetaki, Semir Zeki

**Affiliations:** ^1^ Laboratory of Neurobiology, Department of Cell & Developmental Biology University College London London UK

**Keywords:** artifactual images, biological images, faces, illusory faces, memorability

## Abstract

We have previously suggested a distinction in the brain processes governing biological and artifactual stimuli. One of the best examples of the biological category consists of human faces, the perception of which appears to be determined by inherited mechanisms or ones rapidly acquired after birth. In extending this work, we inquire here whether there is a higher memorability for images of human faces and whether memorability declines with increasing departure from human faces; if so, the implication would add to the growing evidence of differences in the processing of biological versus artifactual stimuli. To do so, we used images and memorability scores from a large data set of 58,741 images to compare the relative memorability of the following image categories: real human faces versus buildings, and extending this to a comparison of real human faces with five image categories that differ in their grade of resemblance to a real human face. Our findings show that, in general, when we compare the biological category of faces to the artifactual category of buildings, the former is more memorable. Furthermore, there is a gradient in which the more an image resembles a real human face the more memorable it is. Thus, the previously identified differences in biological and artifactual images extend to the field of memory.

## INTRODUCTION

Our ability to remember stimuli depends both on our internal states and on external factors which are intrinsic properties of the stimuli. An example of the former is the impoverishment of memorability in general during depressive states (Williams et al., 2000) whereas an example of the latter is the dependence of the memorability of images containing animals on the number of animals in the image (Dubey et al., 2015). Although many other stimulus properties have been examined with regard to their effect on memorability (e.g., Isola et al., 2011), stimuli have not been previously studied for their memorability as a function of the two broad categories to which they belong; namely, the biological and artifactual ones.

We use the term “biological” to refer to non‐man‐made stimuli (e.g., human faces and bodies, trees, flowers, and animals), and the term “artifactual” to refer to man‐made stimuli (e.g., motor vehicles, buildings, and technological devices). Our previous work has shown that there is a difference in the extent to which esthetic judgments for different categories of stimuli are resistant to revision in light of external opinion, with biological stimuli being more resistant than artifactual ones (Bignardi et al., 2021; Chen & Zeki, 2011; Glennon & Zeki, 2021; Zeki & Chén, 2020; Zhang & Zeki, 2022). This distinction has been addressed mainly in the field of visual perception and has not yet been extended to the field of memory.

In this study, we examined memorability for stimuli by concentrating on faces as representative of biological stimuli compared to buildings as representative of artifactual stimuli. We chose our stimuli to represent different categories, beginning with real human faces compared to buildings and progressing through a comparison of real human faces to five categories that differed increasingly from human faces. Previous studies have examined whether there are differences in recognition memory and in neural activity patterns across object categories that differ in their animacy and size (Blumenthal et al., 2018) and differences in neural activity patterns for faces, chairs, and buildings (Martin et al., 2016). We wanted to address the question of how memorability varies, if at all, with stimuli that depart increasingly from resemblance to a real biological stimulus; namely, human faces.

We used a very large number of images derived from a large data set. This has both advantages and disadvantages; the former revolves around the fact that the large number of stimuli includes both the biological and the artifactual categories as well as the subcategories within each, thus making it possible to make a broad comparison. The disadvantage is that the stimuli were not strictly controlled for a number of characteristics such as color, size, angle of view, and so on. We nevertheless hypothesized that if the division between the biological category of faces and artifactual categories is real, a difference in the memorability for human faces versus other categories would emerge even in the absence of strict controls for the factors mentioned.

## METHODS

In all three of our experiments, we used images and memorability scores from the LaMem data set (http://memorability.csail.mit.edu/index.html; Khosla et al., 2015). This data set consists of 58,741 images with memorability scores for each of the images. Each image contains data from 80 Amazon Mechanical Turk workers on average. In Khosla et al.'s (2015) study, each image was presented for 600 ms, and participants were asked whether they had previously seen it during the experiment. Memorability scores can range from 0 (*lowest memorability*) to 1 (*highest memorability*). The memorability score of an image was computed as the hit rate of that image minus the false alarm rate of that image. For each image, we used the mean of the five memorability scores provided in the data set. Five scores were available because Khosla et al. split their data into five parts, each containing the training, validation, and testing sets. To determine the training and validation scores, they used a random half of their subjects and used the other half to find the testing scores.

We inspected carefully the 58,741 images and placed them into different categories depending on our question of interest. We excluded images that showed a word or words. The experimenters in the present study were blind to the previously provided memorability scores. To examine differences in memorability scores between the different image categories, we used Welch's *t* test in the first experiment and Welch's analysis of variance and the Games‐Howell post hoc test in the second experiment. These tests were used because there was no homogeneity of variances, *p* < .001, in Levene's test.

**FIGURE 1:**
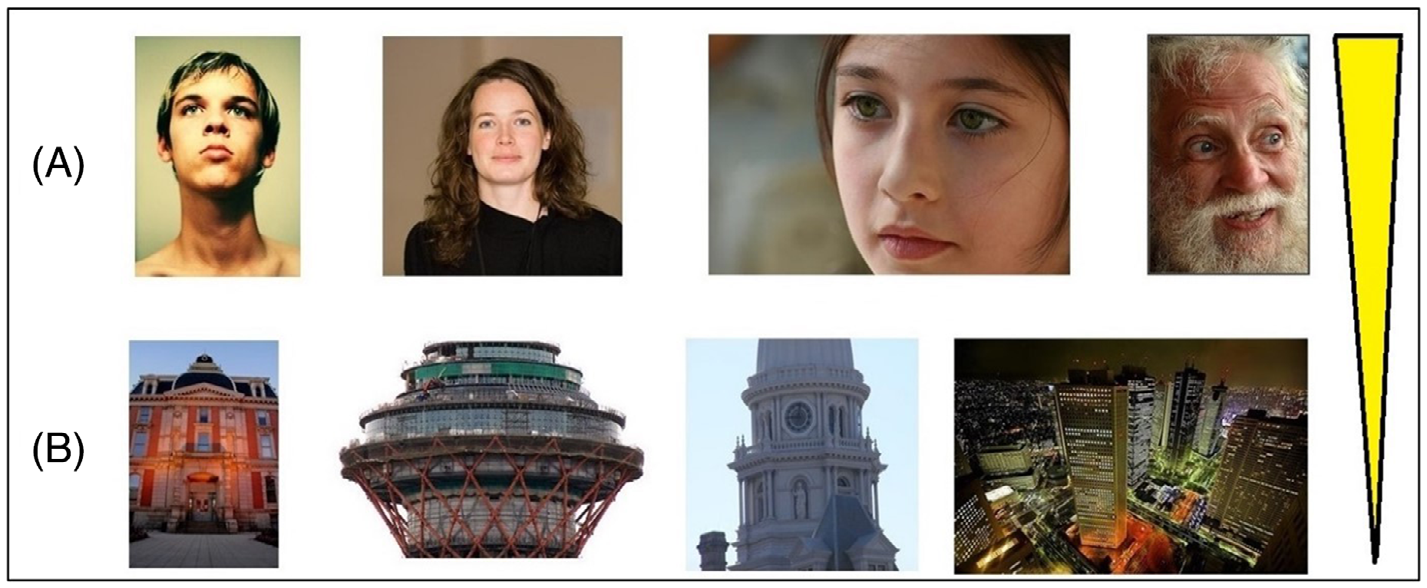
Categories of images analyzed in Experiment 1. (A) Real human faces and (B) buildings. The yellow bar illustrates the increase in memorability from B to A

In our first experiment, we examined the memorability of stimuli depicting only buildings or real human faces (Figure 1) because previous studies examining biological and artifactual stimuli have most often included these types of stimuli. We found 363 images that contained a building or buildings; we excluded images of famous buildings (e.g., Twin Tower building in New York or the Eiffel Tower in Paris). We found 129 images that contained a real human face; here, we excluded images that showed more than one face or showed only parts of a face (e.g., only one eye or only the mouth). No representation of faces or buildings in paintings was used.

**FIGURE 2:**
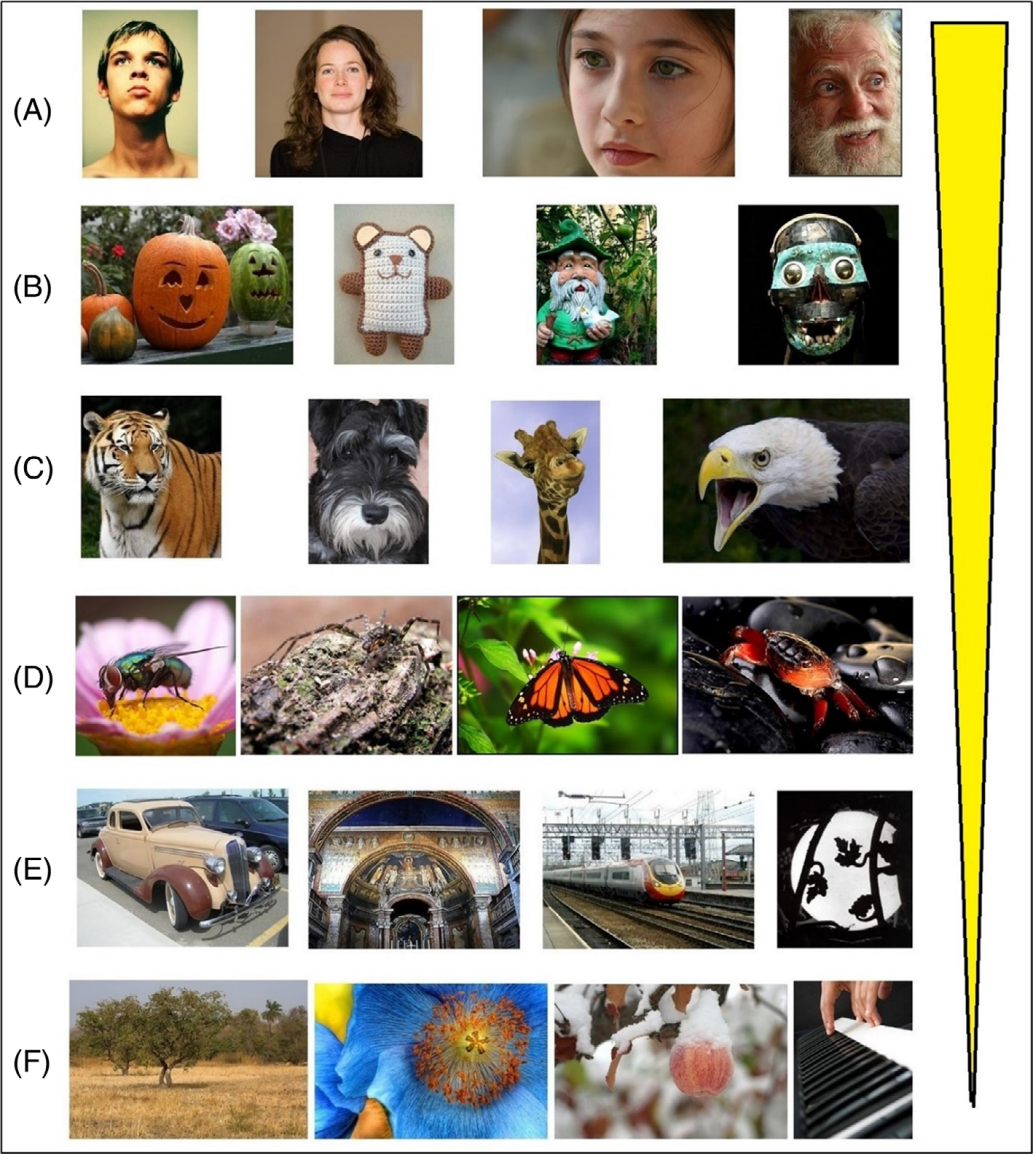
Categories of images analyzed in Experiment 2. (A) Real human faces, (B) face‐like stimuli, (C) animal faces resembling human ones, (D) animal faces with lesser resemblance to human faces, (E) imagined‐face‐like images, (F) non‐face‐like images. The yellow bar illustrates the increase in memorability from F to A

We followed this by comparing the memorability scores for stimuli falling into six different image categories, each successive category departing increasingly from real human faces: real human faces (Figure 2A), face‐like stimuli (Figure 2B), animal faces resembling human ones (Figure 2C), animal faces with lesser resemblance to human faces (Figure 2D), imagined‐face‐like stimuli (Figure 2E), and non‐face‐like images (Figure 2F). For the real human faces category, we used the 129 images from our first experiment. The face‐like category (1,317 images) included replicas of real (animal or human) faces such as those shown in the form of a cartoon image, paintings, sculptures, skulls, dolls, or other toys. It also included examples of face pareidolia (i.e., the perception of illusory faces in random stimuli) in which it was easy to see the face‐like configuration. In the “animal faces resembling human ones” category (2,022 images), we did not include: (a) images that apart from the animal face also contained artifactual stimuli or (b) animals whose head did not resemble a human face (e.g., butterfly, centipede, jellyfish). We had a separate category named “animal faces with lesser resemblance to human faces” (508 images) in which we included images of animals whose head did not much resemble a human face (e.g., butterfly, centipede, jellyfish, snail, spider, bee) and animals whose face could not be clearly seen (e.g., birds shown from a long distance). In the imagined‐face‐like category (9,379 images), we included examples of face pareidolia in which it was difficult to see the face‐like configuration, that is, images that could look like a face if one searched for a long time and/or had some imagination and/or excellent ability to detect faces among many distracting stimuli. In the non‐face‐like category (13,750 images), we included images that did not contain a face and did not look face‐like if seen for a short or long duration.

In our third experiment, we selected the 50 most memorable images and the 50 least memorable ones in the LaMem data set. In that test, the memorability scores for the first category ranged from 0.9894 to 1, and in the latter category ranged from 0.2658 to 0.3644.

## RESULTS

The single most memorable category in our results belongs to real human faces, a result obtained by comparing real human faces against all other categories, *p* < .001, η^2^ = 0.055 (Figure 2 and Table 1) as well as real human faces against buildings (*M* = 0.859, *SEM* = 0.0050 for real human faces; *M* = 0.697, *SEM* = 0.0051 for buildings; *p* < .001, η^2^ = 0.5084, Figure 1). We have noticed that of the 50 most memorable images in the LaMem data set, 32 contained faces or face‐like stimuli; of the 50 least memorable images, only 3 contained a face or face‐like stimuli.

**TABLE 1:** Memorability scores for different image categories

Image categories	*M*	*SEM*	Games Howell test
**Real human faces**	0.859	0.0050	
**All face‐like stimuli (including face‐like artifactual stimuli)**	0.835	0.0021	*p* < .001
**Animal faces resembling human ones**	0.773	0.0021	*p* < .001
**Animal faces with lesser resemblance to human faces**	0.767	0.0044	*p* = .894
**Imagined‐face‐like**	0.732	0.0012	*p* < .001
**Non‐face‐like**	0.718	0.0011	*p* < .001

## DISCUSSION

In this study, we set out to explore whether the previously identified differences in the extent to which esthetic judgments of biological and artifactual images are resistant to updating in light of external opinion (Bignardi et al., 2021; Zeki & Chén, 2020; Zhang & Zeki, 2022) extend to the field of memory. As expected, the largest differences in memorability were found when comparing biological stimuli that were those of real human faces and artifactual ones that were those of buildings. In fact, our results show that there is a gradient in which the more an image looks like a real human face, the more memorable it is (see Figure 2).

Three previous studies (Brady et al., 2019; Dubey et al., 2015; Maguire et al., 2001) have examined memorability for somewhat similar types of stimuli to ours with a different approach and methodology. Maguire et al. (2001) examined memory for buildings, human faces, and animal faces, and Brady et al. (2019) compared memorability for stimuli that had different grades of resemblance to faces (from non‐faces to unambiguous faces). These previous studies, in addition to using only black and white stimuli (unlike our stimuli, most of which were colored) and analyzing a smaller number of images, also presented them for durations that differ from ours, significantly so in the work of Maguire et al. (at 3000 ms) and less so in the work of Brady et al. (at 500 ms). Dubey et al.'s (2015) comparison was between persons and animals, but their stimuli, whether those of humans or animals, were not restricted to their faces. Despite these differences, our results are similar to theirs in finding that memorability scores were higher for persons than for animals. Consistent with the results of Brady et al., we also found that the more the images looked like a face, the more memorable they were.

A clear gradient in memorability, depending on how much images look like a real human face, may depend on several factors: it may be because subjects are more familiar (i.e., have more expertise) with real human faces than with non‐face stimuli; or stimuli that have more self‐related information (e.g., stimuli more like ourselves) are more important than ones containing non‐self‐related information and therefore more demanding of our attention. There seems to be a preference for face‐like stimuli across vertebrate species that is present even at birth and is independent of experience (Di Giorgio et al., 2017; Sugita, 2008). Another possibility is that faces may register more easily in our memory because they possess characteristics such as symmetry. Our eyes tend to look for symmetric structure in the world (Kootstra et al., 2011). This increase in gaze would likely lead to more attention and to greater ability to remember that stimulus (Aly & Turk‐Browne, 2016). Lastly, the judgment of the beauty of faces seems to be more resistant to revision in light of external influences (Bignardi et al., 2021); therefore, when they are encoded, it may be that they are not affected so much by proactive and retroactive interference. It has also been argued that the brain has an inherited, or very rapidly acquired, template for faces and facial configuration, making their recognition easier (Yang et al., 2022; Zeki, 2009).

Functional neuroimaging studies have shown that the fusiform face area and occipital face area play a role in processing both faces and face‐like stimuli (e.g., Liu et al., 2014; Wardle et al., 2020). Importantly, these areas can distinguish human faces from face‐like stimuli and also face‐like stimuli from non‐face‐like stimuli (Wardle et al., 2020). However, there is variability across participants and across illusory face images in the ability to perceive a face‐like pattern and how face‐like this pattern is perceived to be (Liu et al., 2014; Wardle et al., 2022). This variability may have been the reason why there was quite large variability in memorability scores in the imagined‐face‐like category of our second experiment.

We believe that complexity did not affect our results. Gilbert and Schleuder (1990) and Saraee et al. (2020) found that memorability was higher for more complex images. Therefore, according to their results, we would have expected that scenes would be more memorable than were single stimuli such as faces, which is the opposite from what we found.

The limitations of using the LaMem data set are that participant demographics were unavailable, the number of images per category was unbalanced, remember/know responses and confidence ratings were not collected, and it is unknown whether participants were previously familiar with the images shown (i.e., before seeing them in the experiment). Another limitation of our study is that we only examined one modality (vision) and only restricted ourselves to two‐dimensional stimuli within that modality. It has been previously shown that there is a differentiation in memory for real three‐dimensional stimuli versus photographs of real three‐dimensional stimuli (Kapsetaki et al., 2022; Snow et al., 2014). Thus, future studies could examine whether our findings translate to actual stimuli in real life (i.e., real three‐dimensional stimuli rather than images of them) and to stimuli in other modalities (e.g., audition and touch).

## CONFLICT OF INTEREST

The authors declare that there is no conflict of interest to report.

## ETHICS STATEMENT

This study was approved by the Ethics Committee of University College London.

## Data Availability

The data used in this study are freely available online: http://memorability.csail.mit.edu/.
